# Terahertz imaging of human skin pathologies using laser feedback interferometry with quantum cascade lasers

**DOI:** 10.1364/BOE.480615

**Published:** 2023-03-02

**Authors:** Xiaoqiong Qi, Karl Bertling, Mitchell S. Stark, Thomas Taimre, Yung-Ching Kao, Yah Leng Lim, She Han, Blake O’Brien, Angus Collins, Michael Walsh, Jari Torniainen, Timothy Gillespie, Bogdan C. Donose, Paul Dean, Lian He Li, Edmund H. Linfield, A. Giles Davies, Dragan Indjin, H. Peter Soyer, Aleksandar D. Rakić

**Affiliations:** 1 School of Information Technology and Electrical Engineering, The University of Queensland, Brisbane, QLD 4072, Australia; 2 The University of Queensland Diamantina Institute, The University of Queensland, Dermatology Research Centre, Brisbane, QLD 4102, Australia; 3 School of Mathematics and Physics, The University of Queensland, Brisbane, QLD 4072, Australia; 4 Sullivan Nicolaides Pathology, Brisbane, QLD, Australia; 5 School of Electronic and Electrical Engineering, University of Leeds, Leeds LS2 9JT, UK; 6 Department of Dermatology, Princess Alexandra Hospital, Brisbane, Queensland, Australia

## Abstract

Early detection of skin pathologies with current clinical diagnostic tools is challenging, particularly when there are no visible colour changes or morphological cues present on the skin. In this study, we present a terahertz (THz) imaging technology based on a narrow band quantum cascade laser (QCL) at 2.8 THz for human skin pathology detection with diffraction limited spatial resolution. THz imaging was conducted for three different groups of unstained human skin samples (benign naevus, dysplastic naevus, and melanoma) and compared to the corresponding traditional histopathologic stained images. The minimum thickness of dehydrated human skin that can provide THz contrast was determined to be 50 µm, which is approximately one half-wavelength of the THz wave used. The THz images from different types of 50 µm-thick skin samples were well correlated with the histological findings. The per-sample locations of pathology vs healthy skin can be separated from the density distribution of the corresponding pixels in the THz amplitude–phase map. The possible THz contrast mechanisms relating to the origin of image contrast in addition to water content were analyzed from these dehydrated samples. Our findings suggest that THz imaging could provide a feasible imaging modality for skin cancer detection that is beyond the visible.

## 1. Introduction

Skin cancers are the most common groups of cancers diagnosed worldwide, with more than 1.5 million new cases estimated in 2020 [1]. Melanoma, the deadiest form of skin cancer, has an estimated worldwide total of 325 000 new cases and 57 000 deaths in 2020, with the highest incidence rates observed in Australia/New Zealand, followed by Western Europe, North America, and Northern Europe [1]. Melanoma cases are set to increase by 57% by 2040 with an estimated 68% rise in mortality [1]. Currently, the clinical diagnosis of melanoma still requires full skin examination, and if a suspected melanoma is observed using a dermatocope, a skin biopsy/excision is taken followed by histopathologic examination based on visual inspection of lesion morphology using hematoxylin and eosin staining (H&E). Histopathological diagnosis is relatively rapid and has moderate-high reproducibility if there is clear evidence of benign status (naevus) or if there is clear signs of invading melanoma cells through the epidermal-dermal junction (invasive melanoma) [2]. The difficulty lies when there is evidence of tissue architecture disorder and changes in cell morphology which may indicate early signs of a ’dysplastic’ naevus (also benign) or a melanoma *in situ* (malignant but non-invasive). This current diagnostic process has been shown to be neither consistent nor reproducible [2]. The clinical appearance of an early invasive melanoma can often be missed as it may appear small or may have the appearance of a naevus. As a result, some melanomas will not be found until later stages when they have an increased thickness (i.e., more chances for melanoma cells to enter the blood or lymphatic vessels). Early detection of melanoma is still critical to enable curative surgery, however current clinical approaches are unable to fully address this unmet need.

THz radiation lies in between the microwave and infrared regions of the electromagnetic spectrum with a frequency range from 0.1 to 10 THz [3]. It is known as “THz gap” due to the lack of compact and powerful THz laser sources as well as effective THz detectors. However, THz radiation offers great promise for medical and biological imaging applications, such as detection and imaging of cancers [4–6], due to the unique properties of THz waves. These include the high sensitivity to water content and blood flow in tumours, due to the hydrogen bonds present in water [7–10]; the characteristic THz spectral responses of a wide range of molecules, due to the matched frequencies of THz waves with that of molecular vibrations and rotations [11–13]; and the non-ionizing nature of THz radiation owing to the low photon energy (0.4 to 40 meV).

THz contrast in images or dielectric constants has been found in various types of tumours and conditions, including human brain gliomas [14,15], lung cancer [16], breast cancer [17–19], liver cirrhosis [20], colon cancer [21], oral cancer [22], and skin cancers [23–26]. The penetration depth of THz radiation on the skin tissue is 0.1 to 0.3 mm on average [27], which makes it the ideal target for early skin cancer detection. The first THz imaging experiment for skin cancer detection was conducted to distinguish basal cell carcinoma (BCC) from normal tissue in the frequency range of 0.1–2.7 THz [23]. It was found that the tumour tissue had higher refractive index and absorption than that of normal tissue, due to higher water content in the BCC [24]. More recently, the differences in the complex THz dielectric characteristics of dysplastic and non-dysplastic skin naevi were measured *in vivo* in the frequency range of 0.3–1 THz [26].

The majority of the THz studies as mentioned above on biological samples have been performed using a THz time domain spectroscopy (TDS) system. One of the main advantages of THz-TDS systems is their broadband nature which enables sensing over a range of frequencies. The main drawback of THz-TDS systems is the difficulty of generating sufficient power at frequencies beyond 2 THz (thereby limiting both their effective signal-to-noise ratio and their accessible frequency range). Additionally, THz-TDS suffers from slow data acquisition and long scan time caused by the optical delay line. One alternative, but as yet largely unexploited source of THz radiation is the THz QCL [28–33]. The compact size and high emissions power of these sources are particularly well-suited to biomedical imaging systems. Laser feedback interferometry (LFI) is one of the simplest coherent techniques, for which the emission source can also play the role of a highly sensitive detector. The combination of QCLs and LFI is particularly attractive for sensing and imaging applications, notably in the THz band where there is a lack of high-speed high-sensitivity external detectors. Indeed, LFI with THz QCLs – which possess high output power, low phase-noise and stability under optical feedback – has been successfully employed for sensing/imaging and benefits from the intrinsic advantages inherent to homodyning detectors, in particular suppression of unwanted background radiation [34–39]. The use of the QCL itself as the detector means that the detection speed is theoretically limited only by the device itself.

Although THz contrast has been found in various types of tumours, the origin of the contrast mechanism is still not well understood. The difference in the average signal between the normal and tumor regions for the fresh brain tissues was four times larger than those for the paraffin-embedded brain tissues [40]. Preliminary studies estimated that water accounts for only ∼ 50% of the contrast in optical properties between cancerous and healthy cells [41,42]. Importantly, THz waves were found to be sensitive to conformational change of proteins and DNA mutations [4,43–47]. Increases in tumor mutational burden are a hallmark of skin cancers including melanoma. Great attention has been focused on the investigation of intrinsic biomarkers of melanoma at both the protein and DNA level, to aid diagnosis and prognosis and to determine which patients are advised to follow the path of a more aggressive therapy [48–54]. Initial studies have also shown that biomarkers of skin cancer such as tyrosinase (an enzyme for synthesis of melanin) and tryptophan (an intrinsic non-melanoma biomarker) have fingerprints between 2–3 THz [12,13], which aligns with the emission frequency of THz quantum cascade lasers (QCLs).

In this work, we conducted THz imaging for unstained human skin pathologies based on LFI in a narrow band ( ∼ 900 MHz) THz QCL at 2.8 THz. Firstly, the skin sectioning protocols was investigated to achieve both clear contrast between the pathology and surrounding normal skin, and the minimum thickness of the section to reduce sample wastage. Second, using the minimum thickness determined in step 1, we found that even the narrow band THz QCL can be used to obtain the THz contrast for three different types of human skin samples: benign naevi, dysplastic naevi, and melanoma. Finally, we investigated the possible mechanisms of the THz contrast in addition to water content in different skin pathologies. The findings in this work not only identify the feasible methodology of LFI based narrow band THz imaging on differentiating between skin pathologies and normal tissue; it also extends our understanding about the contrast mechanisms contributed to the THz images in addition to the water content.

## 2. Method

### 2.1 Experimental setup

**Fig. 1.**
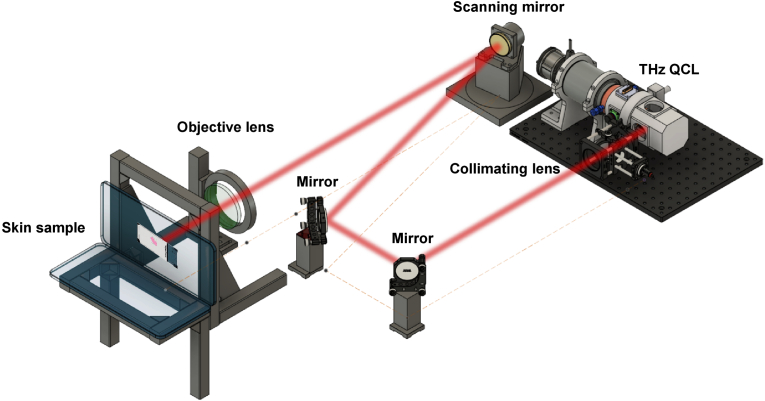
The experimental setup of THz imaging using a THz QCL for human skin samples. The laser beam from the THz QCL is collimated by a collimating lens (focus length: 30 mm and numerical aperture (NA) of 25 mm) and then guided by two mirrors onto a scanning mirror, and then focused by an objective lens (Plano-convex lens LPX-TPX-D50.8-F100, focus length 100 mm and NA 50.8 mm) and incident upon a human skin sample.

The experimental setup of THz imaging using a THz QCL for human skin samples is shown in Fig. 1. The laser beam from a THz QCL is collimated by collimating lens (Tsurupica-RR-CX-1.5-30-SPS, f = 30 mm and d = 25 mm) and then guided by two mirrors onto a scanning mirror before being focused by an objective lens (Tydex LPX-TPX-D25.4-F50, f = 50 mm and d = 25.4 mm) and incident upon a human skin sample. In this typical LFI system, the laser emission from the THz QCL reflects back into the laser cavity from each pixel of the skin sample during scanning process, mixes with the intra-cavity electric field, and generates a measurable self-mixing (SM) signal in terminal voltage of the laser at each skin pixel [36]. The variations in the terminal voltage of the laser, which contains the information about the skin samples, were extracted and amplified as described in [38]. At each pixel of the imaged skin we obtain a time-domain SM signal, which were translated into frequency domain through Fourier transform and provides the spectrum of the interferometric signal, including its amplitude and phase at the frequency of the SM waveform. These amplitude (in dB) and phase (in degree) at each pixel of a skin sample were used to create the THz amplitude and phase images of the sample, respectively. The amplitude and the phase images were then be used to compare with the stained histology image to observe the contrast between the skin pathology and the surrounding healthy skin.

The THz QCL used in the setup consisted of a 12 µm-thick GaAs/AlGaAs 9-well phonon-assisted active region with a design frequency of 2.9–3.2 THz as described in [55]. The structure was grown by solid-source molecular beam epitaxy on a semi-insulating GaAs substrate, with the active region grown between doped upper 50 nm-thick and lower 700 nm thick GaAs contact layers. The wafer was processed into 150 µm wide surface-plasmon ridge waveguide structures using photolithography and wet chemical etching, with the substrate thinned to 200 µm. Devices were then mechanically cleaved to define a ridge of length 1.8 mm. The THz QCL was operated at 50 K by a Stirling cryocooler system and driven by a custom-built laser pulse driver, as described in [38]. The driving current was set as ramped pulse train covering the range from 1.516 A to 1.062 A (454 mA current sweep), in order to produce the LFI signal through tuning the emission frequency from the QCL ( ∼ 900 MHz). The pulse duration is 600 ns and with 14.3% duty cycle.

### 2.2 Sample preparation

There are typically three types of skin tissue that can be used for THz imaging, which are fresh tissue, frozen tissue, and fixed tissue. Although water content in the fresh skin tissue is higher in a cancer tumor which may be observed by THz imaging, water content differences are not only limited to differences between healthy and diseased tissue; it is often seen that fat and muscle tissue also have different THz optical properties [56]. While the problem for frozen samples is the resonances of the frozen crystal will be mistaken as the THz resonances of protein [57]. From this point of view, the fixed tissue potentially allows overcoming these difficulties because the preparation of paraffin blocks includes a tissue dehydration step. This provide a THz imaging method to investigate the THz fingerprints of disease-associated molecular biomarkers apart from the water content.

Institutional approval of experiments involving human tissues was provided by Metro South and The University of Queensland Human Research Ethics Committees (HREC/11/QPAH/363, 2011001201, HREC/17/QPAH/817 and 2018000165). All study participants were recruited from the Princess Alexandria Hospital (PAH) and gave written and informed consent in accordance with the Declaration of Helsinki. Archival tissues used in this study were derived from Sullivan Nicolaides Pathology (Brisbane, Australia) and diagnosed by 3 expert dermatopathologists (BOB, AC, HPS) to provide a consensus diagnosis. The tissues were stored as either Formalin- or PAXgene-fixed, paraffin-embedded (FFPE or PFPE) blocks. Table 1 summarizes the information of 15 excised specimens of human skin for THz imaging, which includes the diagnoses, gender, age, and the centroid data of the skin pathology and surrounding normal skin in the THz phase-amplitude space, respectively (see details in the results section). First of all, two benign naevi blocks from Patient 1 and 2 were used to determine the best experimental protocol of sectioning thickness suitable for THz imaging using QCLs under LFI technology, the details can be found in the Supplement 1. Based on the THz imaging assessment for section thicknesses ranging from 5 µm to 200 µm, it was concluded that the minimum thickness of the skin slice that can still provide the contrast in the THz amplitude and phase images is 50 µm, namely, one half-wavelength of the THz wave used. After that, two compound naevus blocks from Patient 3 and 4, two dysplastic naevus blocks from Patient 5 and 6, and nine melanoma blocks from Patient 7 to 15 were sectioned into 50 µm and imaged by our THz imaging system. We used polystyrene microscope slides of 3 × 1 with an thickness of 1 mm to load the sectioned slice for THz imaging due to their low absorption in the THz range compared to other standard polymers [17]. Between each tissue section, a 5 µm-thick sectioning was conducted and mounted on a standard glass microscope slide, and subjected to staining with H&E for the purpose of pathology assessment and correlation to the THz images. No staining or any contrast agents were used to the sections used for THz imaging.

**Table 1.** Study cases with the centroid data of each skin pathology and surrounding normal skin in the THz phase-amplitude space.

Patient number	Diagnosis	Gender	Age at diagnosis	Normal (THz)	Skin pathology (THz)
1	Benign naevus (PFPE)	Male	51	(306°, 0.9992)	(308.3°, 1.121)
2	Benign naevus (PFPE)	Male	42	(60.77°, 1.099)	(156.5°, 1.525)
3	Compound naevus (FFPE)	Male	35	(202.7°, 0.994)	(132.8°, 1.201)	
				(269.5°, 1.004)	(98.7°, 1.253)
4	Compound naevus (FFPE)	Female	50	(255°, 1.026)	(118.9°, 1.224)
5	Dysplastic naevus (FFPE)	Male	28	(245°, 1.066)	(280.4°, 1.293)
6	Dysplastic naevus (FFPE)	Male	33	(218.8°, 0.9906)	(272.9°, 1.187)
7	MM level III (PFPE)	Male	65	(327.4°, 1.032)	(69.36°, 2.788)
8	MIS (PFPE)	Male	76	(32.27°, 1.07)	(274°, 1.959)
9	MM level II (PFPE)	Male	80	(164.1°, 1.269)	(272.1°, 2.226)
10	MIS- lentigo maligna type (PFPE)	Male	NA	^*a*^	
11	MIS- lentigo maligna type (PFPE)	Male	NA	(244.4°, 1.098)	(280.1°, 1.366)	
12	MM (PFPE)	Female	NA	^*b*^	
13	Lentigo MM level II (PFPE)	Female	90	^*c*^	
14	Lentigo MM (PFPE)	Female	NA	^*d*^	
15	Lentigo MM (PFPE)	Female	NA	(311.5°, 0.9773)	(322.6°, 1.343).

^*a*^ Histology review indicated no presence of melanoma on the sectioned slice

^*b*^ Histology review indicated only few melanocytes on the sectioned slice

^*c*^ Histology review indicated the requirement for re-excision

^*d*^ Histology review indicated the size of the melanoma region < 100 μ m, not resolvable by the THz imaging

### 2.3 maging and analysis procedures of the human skin samples

We demonstrate THz imaging results for three different groups of sectioned human skin samples (two patients from each group), including benign naevus, dysplatic naevus, and melanoma. We used the same sample analysis procedure for all the skin samples. First of all, the H&E stain image of the sample was assessed by dermatopathogist (HPS). The diagnosis and classification of the sample (as listed in Table 1) was provided together with the marked regions of the pathology and normal skin in the H&E stain image. We then used the stained images with the marked regions as the mask to find the corresponding regions in the THz images. Next, we overlaid the H&E stain image with the marked regions and the THz amplitude image (both images are visible by setting the opacity of the stained image as 70%). We were then able to change the size of the THz images with fixed aspect ratio and the orientation of the stained images together with the markers to align them in the skin outline. Once aligned, the mask image (H&E stain image) was deleted which left only the THz amplitude image with the marked regions of pathology and normal skin. We repeated the same process to the THz phase image to find the corresponding regions there. The microscope photo of each sectioned sample for THz imaging was also obtained and shown as a reference in the results. Furthermore, the clustering of the pixels in the marked pathology area and normal skin area was analyzed by density distribution method and plotted in the amplitude–phase space, to test if the pathology region can be separated from the normal skin from the same sample in the amplitude–phase space. The THz amplitude of the pathology region was normalized by the mean value of the THz amplitude of the normal skin, then the point cloud was obtained using the pixels in the skin pathology region and the surrounding healthy region selected in the THz images based on the histology review results. After that we calculated the centre of gravity of the point cloud by applying the Mean-Shift algorithm iteratively with a Gaussian kernel [58] for all the samples, as summarized in Table 1.

## 3. Results

### 3.1 THz imaging results for benign naevus samples

**Fig. 2.**
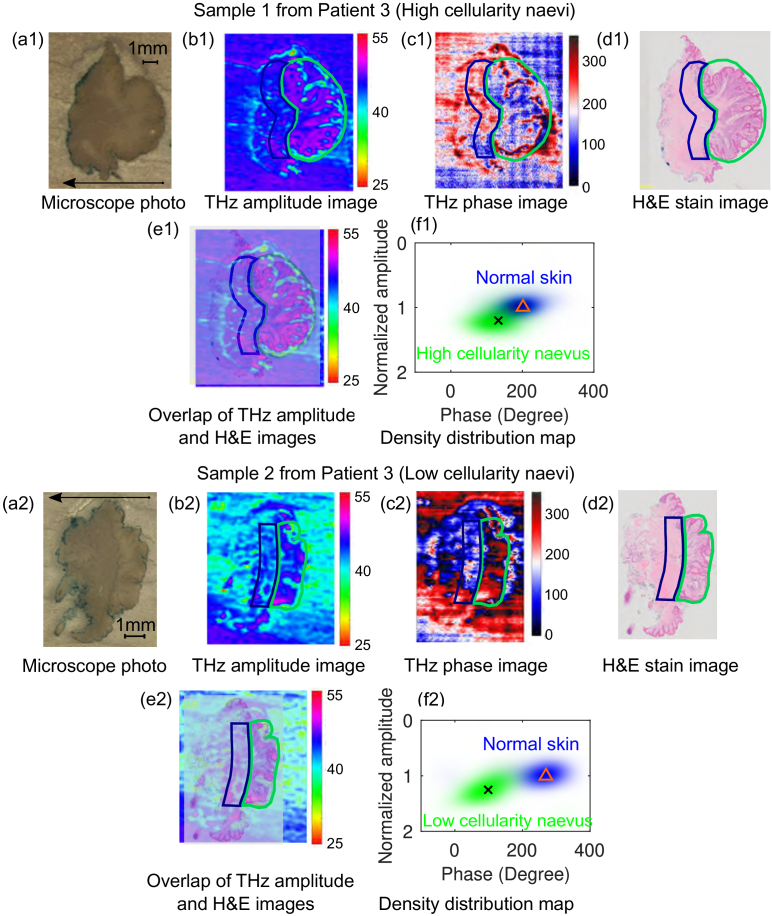
Comparison of THz images with the microscope photo and histology image for a compound naevus (Patient 3) that contain high cellularity naevus cells (top) and low cellularity naevus cells (bottom): (a1) and (a2): Microscope photo (the arrow indicates the direction from the epidermis to dermis layer of the skin sample); (b1) and (b2): THz amplitude image; (c1) and (c2): THz phase image; (d1) and (d2): H&E stain image; (e1) and (e2): Overlap of the THz amplitude and H&E images; (f1) and (f2): Density distribution of the normal skin and naevus in the THz amplitude and phase map, along with the centroids: (202.7°, 0.994) and (132.8°, 1.201) for normal skin and high cellularity naevus region, (269.5°, 1.004) and (98.7°, 1.253) for normal and low cellularity naevus region. The circled region in green and dark blue in (b1) to (e1) and (b2) to (e2) indicate the naevus cell region and normal skin region, respectively.

In this section we provide THz imaging results for two benign naevi samples from Patient 3 and 4. Firstly, we conduct THz imaging for a pair of compound naevus sample sectioned from the same Patient 3. The microscope photo of these two sections are shown in Fig. 2(a1) and (a2), respectively. Sample 1 contains high cellularity naevus cells (as circled by green lines in Fig. 2(b1)–(e1)) and Sample 2 contains low cellularity naevus cells (as circled by green lines in Fig. 2(b2)–(e2)). The normal skin on the left side of the naevus used for comparison is circled by dark blue box in these images. It is interesting to note that similar cellularity structure as observed in the circled area in the stained image [Fig. 2 (d1)] can be clearly observed in the THz amplitude image [Fig. 2(b1)] as well. However, this structure is invisible from its microscope photo [Fig. 2(a1)]. The contrast between high cellularity naevus cells and the normal skin region is also obtained from the THz phase image [Fig. 2(c1)]. The THz amplitude from the circled naevus region in Sample 2 is lower than that from Sample 1, likely due to the lower cell density in Sample 2 [24]. However, good contrast between the low cellularity naevus region (green circled region) and the normal skin region (blue circled region) can still be observed in the THz amplitude and phase images [Fig. 2 (b2) and (c2)]. The density distribution map for Sample 1 and 2 shown in Fig. 2 (f1) and (f2) further demonstrate the separation of the naevus cells and surrounding normal skin in the amplitude–phase space. In order to compare between these two samples, the whole phase values for each of the sample are shifted so that the normal skin are both located around 200°. It can be observed that the relative distribution of the naevus cell (green cloud) and normal skin region (blue cloud) for Sample 1 and 2 are very similar, which means that the reflection from both high and low cellularity naevus cell region is higher than that from its own normal skin region and the phase of the centroids are also separated.

**Fig. 3.**
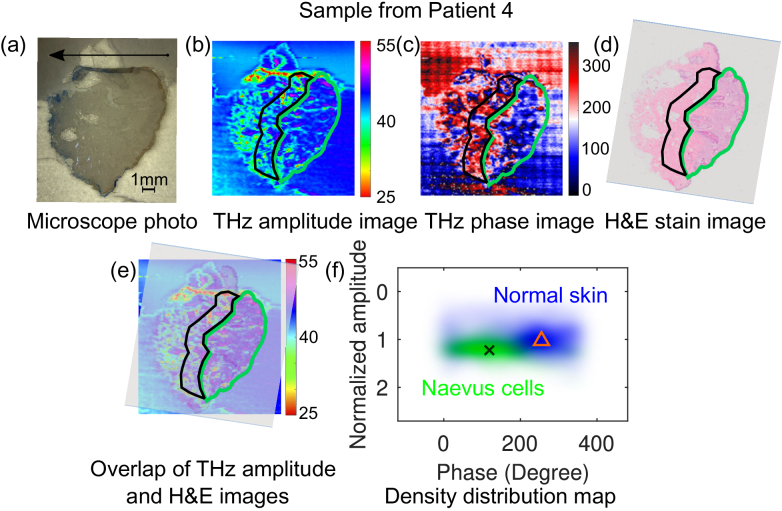
Comparison of THz images with the microscope photo and histology image for a compound naevus from Patient 4: (a) Microscope photo (the arrow indicates the direction from the epidermis to dermis layer of the skin sample); (b) THz amplitude image; (c) THz phase image; (d) H&E stain image; (e) Overlap of the THz amplitude image and the H&E stain image; (f) Density distribution of the normal skin (pixels in the blue box) and naevus cells (pixels circled by the green line) in the THz amplitude and phase map, along with the centroids: (255°, 1.026) for normal skin and (118.9°, 1.224) for the naevus cells region. The circled region in green and blue in (b) to (e) indicate the compound naevus cell region and normal skin region, respectively.

Figure 3 shows a comparison of the THz images for another sample of compound naevus from Patient 4 together with its microscope photo and the corresponding H&E stain image. It is hard to see clear contrast in the microscope photo [Fig. 3(a)]. However, the THz contrast between the naevus region (green circled region) and the normal skin (in the blue box) can be observed from both amplitude and phase images [Fig. 3(b) and (c)]. This contrast agrees very well with the corresponding marked regions in the H&E stain image [Fig. 3(d)], where the green circled region contains the naevus cells and quite a few sebaceous glands while the blue box indicates the normal skin region. The marked regions for the normal skin and naevus cells were used to generate the density distribution in the THz amplitude and phase map as shown in Fig. 3(f), where the triangle and cross indicate the centroids of the normal skin and the naevus cell region, respectively. They are separated in both amplitude (1.026 and 1.224 for the normal skin and naevus cells respectively) and in the phase (255° and 118.9° for the normal skin and naevus cells respectively). It should be noted from comparison of the microscope photo [Fig. 3(a)] and H&E image [Fig. 3(d)] that the top edge of the THz skin slice has been folded during the sectioning and loading process. Moreover, there is a thinner layer of triangle-shape paraffin connected to the top edge of the THz skin slice. These lead to the slice shape difference between the THz and the stained slices, which has to be borne in mind when align them in Fig. 3(e). We used the overall curvature of the skin on the right side when align these two images, so we can ignore the mismatch on top of the slices.

### 3.2 THz imaging results for dysplastic naevus samples

**Fig. 4.**
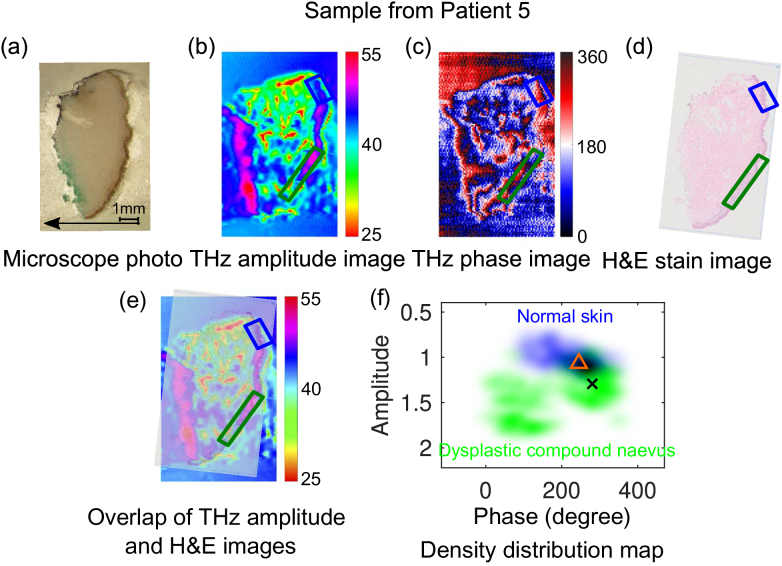
Comparison of THz images with the microscope photo and histology image for a dysplastic naevus tissue from Patient 5: (a) Microscope photo (the arrow indicates the direction from the epidermis to dermis layer of the skin sample); (b) THz amplitude image; (c) THz phase image; (d) H&E stain image; (e) Overlap of the THz amplitude image and H&E images; (e) Density distribution of the point cloud together with the centroid for the normal skin (245°, 1.066) and dysplastic compound naevus (280.4°, 1.293) in the THz amplitude–phase map, indicated by the blue and green clouds, and triangle and cross markers, respectively. The green and blue boxes in (b) to (e) indicates the dysplastic compound naevus cell region and normal skin region, respectively.

**Fig. 5.**
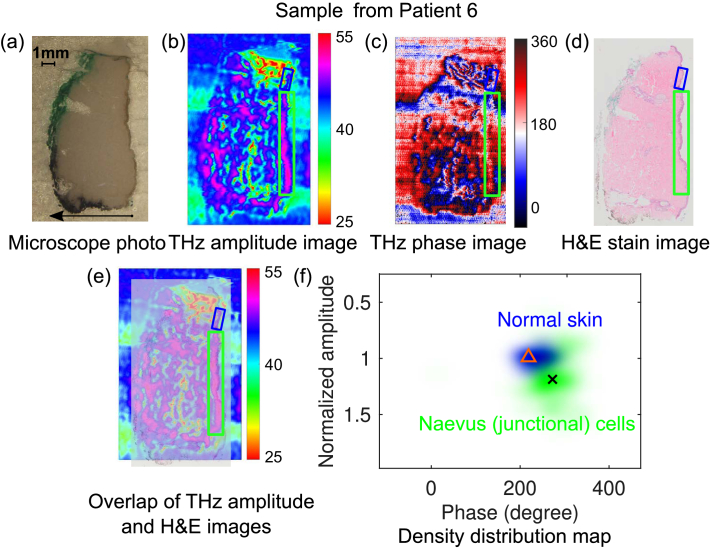
Comparison of THz images with the microscope photo and histology image for a dysplastic naevi tissue from Patient 6: dysplastic naevus tissue from patient 6: (a) Microscope photo (the arrow indicates the direction from the epidermis to dermis layer of the skin sample); (b) THz amplitude image; (c) THz phase image; (d) H&E stain image; (e) Overlap of the THz amplitude and H&E images; (f) Density distribution of the point cloud together with the centroid for the normal skin (218.8°, 0.9906) and dysplastic naevus (272.9°, 1.187) in the THz amplitude and phase map, indicated by the blue and green clouds, and triangle and cross markers, respectively. The marked region in green and blue boxes in (b) to (e) indicates naevus cell region and normal skin region, respectively.

Epidemiological data suggest that high numbers of dysplastic naevus are associated with higher risk of melanoma [59]. In this section, we conducted THz imaging for two dysplastic naevus samples (Sample 5 and 6) and the results are shown in Fig. 4 and Fig. 5, respectively.

Figure 4 compares the THz images for a dysplastic naevus sample from Patient 5 with the microscope photo and the H&E stain image. The microscope photo of the section used for THz imaging is shown in Fig. 4(a). According to the final diagnosis based on the histological image shown in Fig. 4(d), the lower part of the epidermis layer contains dysplastic compound naevus cells, as marked in the green box, and the normal skin in the upper part of the sample used for comparison is marked in the blue box. The THz amplitude and phase image of this sample are shown in Fig. 4(c) and (d), respectively. It can be observed that the marked region in the THz amplitude image contains mixture of high amplitude (purple) and low amplitude (light blue) regions. However, the amplitude for the normal skin (blue) is in between. Similar observations are obtained in the THz phase image. This leads to two separate green clusters of the pixels in the green box in the THz amplitude–phase space, centered around 100° and 280°[Fig. 4(f)]. But the centroid indicates the amplitude of the overall dysplastic naevus is 1.293, marked by the cross, which indicates higher reflections from the dysplastic naevus than the normal epidermis skin with the centroid of amplitude at 1.066, marked by the triangle in Fig. 4(f).

As another dysplastic naevus example, the sample from Patient 6 was sectioned and imaged by the THz imaging system, and the results are shown in Fig. 5. Good contrast between the naevus (junctional) cell region marked in the green box and the normal skin indicated in blue box is observed in both THz amplitude and phase images [Fig. 5(b) and (c)], which agrees well with the stained image [Fig. 5(d)]. Similarly, by combing the THz amplitude and phase images, the density distributions of the pixels in the normal skin (blue cloud) and dysplastic naevus (green cloud) in the THz amplitude–phase space are shown in Fig. 5(f). It can be observed that the junctional naevus region also have higher reflection to the THz radiation around 2.8 THz than the normal skin, with the centroids indicating the amplitude for normal skin and naevus (junctional) are 0.9906 and 1.187, respectively. The two regions are also separated in the phase in Fig. 5(f), with the centroids showing the phase value of 218.8° and 272.9° for the normal skin and naevus cells.

### 3.3 THz imaging results for melanoma samples

**Fig. 6.**
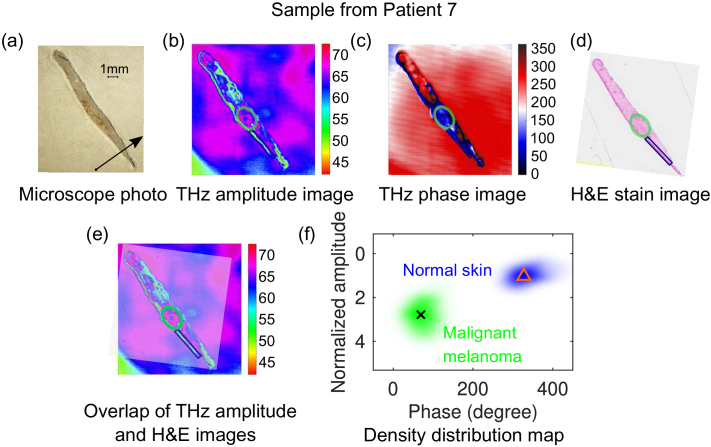
Comparison of THz images for a melanoma sample from Patient 7 with the microscope photo and H&E stain image: (a) Microscope photo (the arrow indicates the direction from the epidermis to dermis layer of the skin sample); (b) THz amplitude image; (c) THz phase image; (d) H&E stain image; (e) Overlap of THz amplitude and H&E images; (f) Density distribution of the point cloud together with the centroid for the representative normal skin at lower epidermis and melanoma cells in the THz amplitude and phase map, indicated by the blue and green clouds, triangle and cross markers at (327.4°, 1.032) and (69.36°, 2.788), respectively. The circled region in green and blue in (b) to (e) indicate the malignant melanoma cells and normal skin, respectively.

Apart from benign naevus and dysplastic naevus samples, we also conducted THz imaging for two melanoma samples and the results are demonstrated in this section. Figure 6 demonstrates the comparison of THz images for the first melanoma sample from Patient 7. The microscope photo of the THz slice together with its THz images are shown in (a), (b) and (c), respectively. Pathologically, the tissue exhibits invasive melanoma spread from epidermis into dermis layer of the skin in the circled green area [Fig. 6(d)], so it was diagnosis as malignant melanoma (MM) level III as shown in Table 1. The normal skin region as marked in the blue box by HPS in Fig. 6(d). Both pathology location and normal skin regions are mapped to the THz amplitude and phase images through overlapping process shown in Fig. 6(e). Interestingly, significant contrasts between the MM (circled in green) and the normal skin (circled in blue) can be observed from both THz amplitude and phase image, as shown in Fig. 6 (c) and (d). The calculated density distribution of the point cloud for the normal skin and MM in the THz amplitude–phase map is shown in Fig. 6(f), where the normal skin indicated by the blue cloud is totally separated with the MM region shown by green cloud. The centroids for these two clusters are indicated by the triangle marker at (327.4°, 1.032) and the cross marker at (69.36°, 2.788). The mean value of the THz reflection from the MM region is 2.7 times higher than that from its normal skin.

**Fig. 7.**
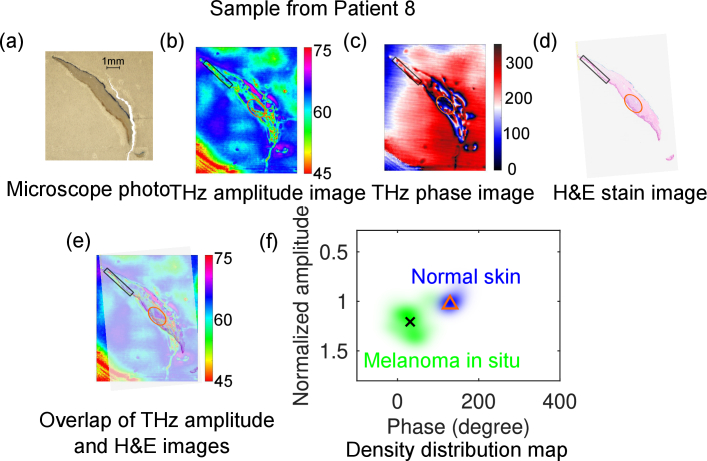
Comparison of THz images for a melanoma sample from Patient 8 with the microscope photo and H&E stain image: (a) Microscope photo (the arrow indicates the direction from the epidermis to dermis layer of the skin sample); (b) THz amplitude image; (c) THz phase image; (d) H&E stain image; (e) Overlap of THz amplitude and H&E images; (f) Density distribution of the point cloud together with the centroid for the representative normal skin at lower epidermis and melanoma cells in the THz amplitude and phase map, indicated by the blue and green clouds, triangle and cross markers at (32.27°, 1.07) and (274°, 1.959), respectively. The circled region in green and blue indicate the melanoma (*in situ*) cells and normal skin, respectively.

The discrimination of THz imaging on melanoma tissue was further tested on a second sample from Patient 8. The results can be found in Fig. 7. This patient was diagnosed as melanoma *in situ* (MIS) with the melanoma cells and normal skin marked in green and blue in the stained image in Fig. 7 (d), which is not visible in the microscope photo shown in Fig. 7(a). The melanoma cells provide higher reflection in the THz amplitude image, as shown in the corresponding mapped regions in Fig. 7(c) after the overlapping process in (e) with (d) as the mask. This contrast was also observed in the THz phase image shown in Fig. 7(d). Similar analysis of the density distribution map for the MIS and normal skin is presented in Fig. 7(f). The normal skin and the MIS cells are indicated by blue and green cloud, respectively, with the centroids for each region indicated by triangle and cross markers at (32.27°, 1.07) and (274°, 1.959), respectively. Similar to the clustering result for melanoma sample 1, both amplitude and phase can be totally separated in the THz amplitude–phase map, but the centroid amplitude ratio between the MM and its normal skin obtained from Fig. 6 (2.7) is higher than that between the MIS and the normal skin (1.8). This means the more advanced the melanoma stage is, the higher THz reflection from the melanoma region. This can be observed from the centroid data from most of the samples as summarized in Table 1. The normalized THz amplitudes from naevi is mostly smaller than 1.3 (Patient 1, 3, 4, 5, and 6), while that from melanoma samples is usually larger than 1.3 (Patient 7, 8, 9, 11, and 15).

## 4. Discussion

Most of the THz imaging for biological samples used THz TDS systems with low power emission on the order of µW and very low signal-to-noise ratio above 2 THz. THz TDS systems have the distinct advantage of broadband operation, which permits its use in spectroscopy, while THz QCL has the important advantages of high-power emission at narrow band frequencies above 2 THz. Here we report THz imaging experiments for human skin samples by using a narrow band ( ∼ 900 MHz) THz source around 2.8 THz. The minimum thicknesses of the dehydrated tissue section to provide the THz contrast from both broadband and narrow band THz radiation sources are on the order of tens of µm. For example, THz contrast was reported in 20 µm and 30 µm-thick slices of fixed human lobular carcinoma and infiltrating ductal carcinoma with surrounding healthy tissue with pulsed THz imaging and spectroscopy system [17]. We demonstrated that by using the coherent LFI technology, even with a narrow band THz QCL, good THz images (amplitude and phase) that are highly correlated with corresponding conventional histology images can be obtained for three different types of pathology skin samples, including benign naevus, dysplastic naevus, and melanoma. Compared with conventional THz imaging technology based on broadband sources, QCL based THz imaging provide higher emission power ( ∼ mW) and higher spatial resolution around the diffraction limitation ( ∼ 110 µm). We found that the skin pathologies can be separated from the surrounding normal healthy skin on the THz amplitude–phase space, meaning that both THz amplitude and phase of the interferometric signal are required for the separation. The amplitude of the signal is determined by the reflectivity from the sample while the phase of this signal contains the phase shift on the reflection from the sample. The reflectivity and phase shift from a target are in turn determined by the complex refractive index of the target [60].

In principle, the contrast in the THz amplitude and phase images originated from different reflectivity and phase shift of the interferometric signal from the sample pixels at the particular emission laser frequency in our technology. It was observed here that for healthy skin the epidermis layer usually has higher THz reflection than the dermis layer. This could be due to different skin constitutes in these two layers. As the outermost layer of the skin, the epidermis layer is mainly composed of melanin cells, keratinocytes, and basal cells in human, while the dermis constitutes of collagen, fat, fibres, blood vessels, sweat glands, and hair follicles.

**Fig. 8.**
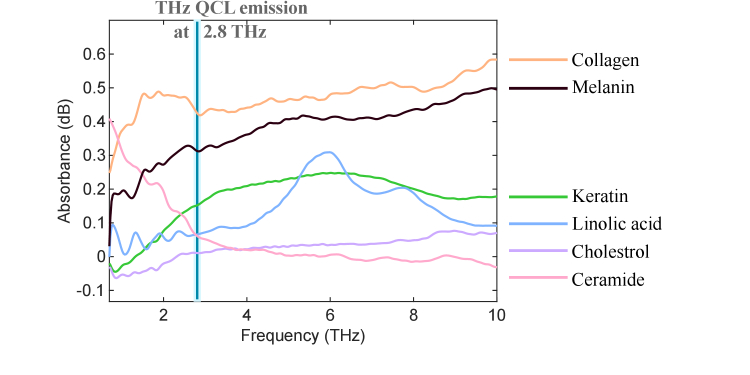
Absorption of basic skin constituents in the THz band (0.6–10 THz), including collagen, melanin, keratin, linolic acid, cholestrol, and ceramide. The narrow band THz QCL emission at 2.8 THz that was used in this work is also shown in the figure.

We measured the absorption spectrum of the basic skin constituents purchased from Sigma-Aldrich at Australian Synchrotron, including collagen, melanin, keratin, linolic acid, cholesterol, and ceramide. We mixed each of the skin constitute powder with pure paraffin (Australian wax Company) with the weight ratio of 5% (the total weight of each pellet is approximately 40 mg). The mixed powder was then made into pellets (7 mm in diameter) to mount onto transmission mode synchrotron sample holder. The pure paraffin pellet was used as reference to normalize the transmission spectrum of the skin constitutes to obtain the transmittance. The absorbance (in dB) of each skin constituent was calculated using –log10 (transmittance), according to the Beer-Lambert Law. The absorbance of basic skin constituents are shown in Fig. 8. It can be observed that at the narrow band THz QCL emission frequency around 2.8 THz, collagen (the main skin constituent in dermis layer) has stronger absorption than melanin and keratin (the dominant constituents of the epidermis layer). The THz contrast observed in the dermis layer is likely due to varying content of fatty tissue, nerve fibre, or fibrous connecting tissue with lymph and blood capillaries. In addition, the THz amplitude from three different pathologies is always higher than its own healthy skin, which indicates less absorption and higher reflection of the THz radiation from the pathology locations. Apart from that, different level of cellularity can also contribute to the THz contrast in the amplitude and phase images. In particular, we found that the high cellularity structure can be observed in the THz images, which indicates the origin of THz contrast from varying cell densities, the higher the cell density, the stronger the THz amplitude. It was reported by Wallace et al. [24] that the cellularity and collagen or mucin content may reduce the percentage of water for freshly excited softy tissue, which will reduce the THz absorption and increase the reflection in the fresh tissue. And here for dehydrated tissue, we also observe higher reflections from the higher density cells due to less absorption. However, the THz contrast between pathology and healthy skin in dehydrated tissue is opposite to that in fresh tissue with water content. In dehydrated tissue samples, the pathology locations have higher reflection than surrounding normal skin while in a fresh sample, the pathology locations have lower reflection than the surrounding normal skin due to strong water absorption in tumors. But water is not the sole source that contributes to THz contrast in skin cancer tissues.

**Fig. 9.**
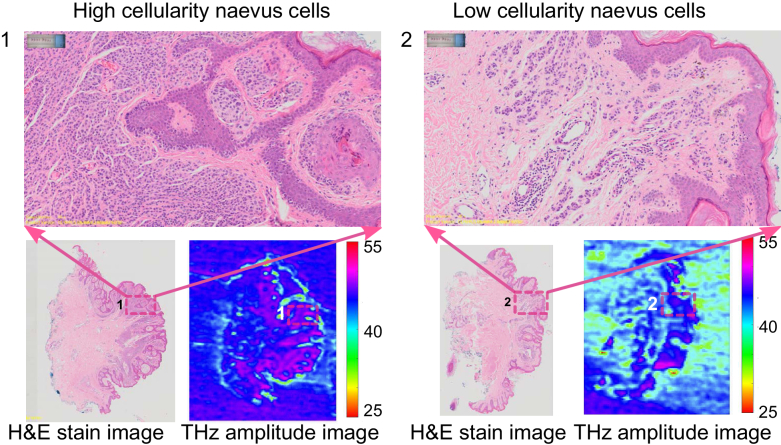
Comparison of zoomed in H&E stain image and the THz amplitude image: 1 High cellularity naevus cells from Sample 1 of Patient 3; 2 Low cellularity naevus cells from Sample 2 of Patient 3.

**Fig. 10.**
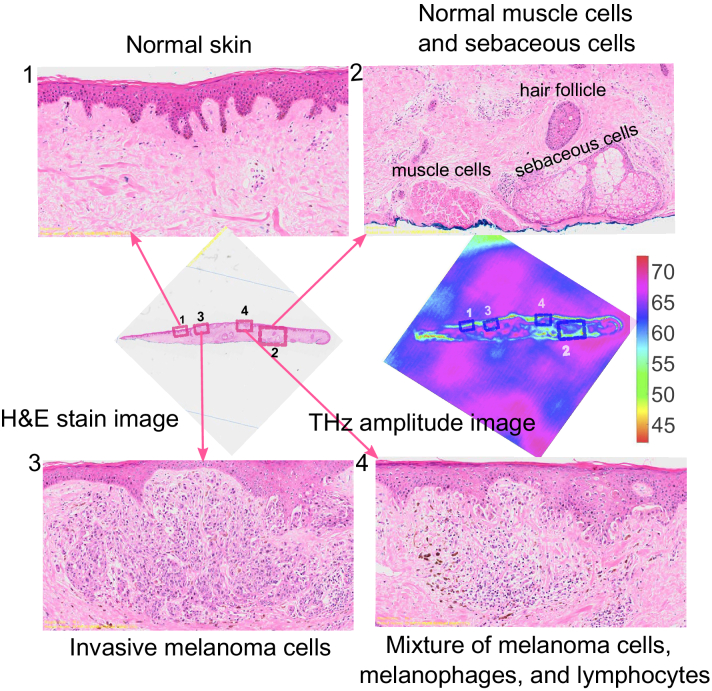
Comparison of zoomed in H&E stain image and the THz amplitude image for the sample from Patient 7: 1 Normal skin at Position 1; 2 Normal muscle cells and sebaceous cells at Position 2; 3 Invasive melanoma cells at Position 3; 4 Mixture of melanoma cells, melanophages and lymphocytes at Position 1.

To better understand the relationship between cellularity and THz contrast, we compared the THz amplitude between two regions with known naevus cell cellularities in Fig. 9. The naevus cells are small and purple-stained, Position 1 contains more densely-packed cellular naevus cells as opposed to Position 2. The contrast between Postion 1 and 2 can be observed in the THz amplitude images as well, where the THz amplitude at Position 1 corresponding to the high cellularity naevus cells shows higher reflection than that at Position 2 with the low cellularity naevus cells. For melanoma samples, different amounts of melanoma cells also contribute to the THz contrast in the images. The comparison of zoomed in pathology between normal skin and the skin area containing melanoma cells are presented in Fig. 10. In particular, Position 2 contains smooth muscle cells, sebaceous cells, and hair follicles as labeled, which corresponding higher THz amplitude than that of Position 1, as shown in the THz amplitude image. Position 3 includes purple-stained melanoma cells and Position 4 contains a mixture of melanoma cells, melanophges, and lymphocytes as shown in 3 and 4 in Fig. 10, respectively. Correspondingly, the THz amplitude at the melanoma cell region (Position 3 and 4) shows higher reflection, when compared with that in the normal skin region (Position 1).

It should be noted that for the ex-vivo study, apart from the sample itself, many other factors can also contribute to the THz contrast, which includes air bubbles at the boundary of the sample and the surrounding paraffin or cracking of the paraffin, and the folding of the skin at the edge of the slice. Nevertheless, these factors can be excluded from the microscope photo of the sample slice used for THz imaging. Therefore, it is very important to compare the microscope photo with the THz images as well as the histology stained image during the image analysis.

## 5. Conclusion

In conclusion, we demonstrated that a narrow band THz QCL based laser feedback interferometry technology at 2.8 THz can be used to observe THz contrast between different pathologies and surrounding healthy skin of unstained dehydrated human skin samples. We identified the minimum thickness of the dehydrated human skin section that can provide THz contrast is one half-wavelength of the narrow band THz emission frequency from the laser source. We obtained high-resolution THz amplitude and phase images from three different types of human skin samples (benign naevus, dysplastic naevus, and melanoma) at the diffraction limited spatial resolution. Proof-of-concept experiments conducted on these three different types of the human skin samples demonstrated identifiable contrast in THz imaging which correlated well with the histopathological stain identification. The locations of pathology and surrounding healthy skin can be separated in the THz amplitude–phase map. The observed contrast in the THz images of skin samples is a combination of contrast mechanisms which include different constituents of the epidermis and dermis layer, varying cell densities and structures, different content of melanoma cells, and other artificial factors induced by the sample preparation process. Since THz reflections from the epidermis layer is higher than that from the dermis layer, and it is not always possible to choose the disease and normal skin in the same layer (such as for raised naevus, the epidermis layer is dominated by the disease skin, we can only choose the normal skin in the dermis layer as a reference, which increases the normalized THz amplitude), comparison among different skin pathologies should build on normalization the pathology values by the normal skin cross different layers and from larger number of samples. A large number of samples of the THz images also allows for training and testing by using statistical analysis based on machine learning algorithms for the purpose of skin cancer diagnosis. On the other hand, fully understanding the contributions of the molecular biomarkers of skin cancer to the contrast of THz images is a potential challenge and the key element for applying THz imaging in clinical detection. Furthermore, non-invasive detection requires *in vivo* THz imaging, where the THz contrast will be associated with a change in water content in the skin tissue. Understanding the contributions of specific biomarkers of skin cancer to the *in vivo* THz images is also very important for the practical application of THz imaging to skin cancer detection.

## Data Availability

Data underlying the results presented in this paper are not publicly available at this time but may be obtained from the authors upon reasonable request.

## Supplemental document

See Supplement 1 for supporting content.
