# Supplementary material for: Terahertz imaging of human skin pathologies using laser feedback interferometry with quantum cascade lasers
Source: Biomed Opt Express. 2023 Mar 2;14(4):1393–410. doi: 10.1364/BOE.480615 (PMC10110320; doi:10.1364/BOE.480615)
Supplement: Supplementary file 1 [file boe-14-4-1393-s001.pdf]

Supplemental Document

Biomedical Optics EXPRESS

Terahertz imaging of human skin pathologies using laser feedback interferometry with quantum cascade lasers: supplement

XIAOQIONG QI,[1] KARL BERTLING,[1] MITCHELL S. STARK,[2] THOMAS TAIMRE,[3] YUNG-CHING KAO,[2] YAH LENG LIM,[1] SHE HAN,[1] BLAKE O'BRIEN,[4] ANGUS COLLINS,[4] MICHAEL WALSH,[4] JARI TORNIAINEN,[1] TIMOTHY GILLESPIE,[1] BOGDAN C. DONOSE,[1] PAUL DEAN,[5] LIAN HE LI,[5] EDMUND H. LINFIELD,[5] A. GILES DAVIES,[5] DRAGAN INDJIN,[5] H. PETER SOYER,[2,6] AND ALEKSANDAR D. RAKIĆ[1,*]

[1]*School of Information Technology and Electrical Engineering, The University of Queensland, Brisbane, QLD 4072, Australia*
[2]*The University of Queensland Diamantina Institute, The University of Queensland, Dermatology Research Centre, Brisbane, QLD 4102, Australia*
[3]*School of Mathematics and Physics, The University of Queensland, Brisbane, QLD 4072, Australia*
[4]*Sullivan Nicolaides Pathology, Brisbane, QLD, Australia*
[5]*School of Electronic and Electrical Engineering, University of Leeds, Leeds LS2 9JT, UK*
[6]*Department of Dermatology, Princess Alexandra Hospital, Brisbane, Queensland, Australia*
[*]*rakic@itee.uq.edu.au*

This supplement published with Optica Publishing Group on 2 March 2023 by The Authors under the terms of the Creative Commons Attribution 4.0 License in the format provided by the authors and unedited. Further distribution of this work must maintain attribution to the author(s) and the published article's title, journal citation, and DOI.

Supplement DOI: https://doi.org/10.6084/m9.figshare.22068140

Parent Article DOI: https://doi.org/10.1364/BOE.480615

Supplemental document: Terahertz imaging of human skin pathologies using laser feedback interferometry with quantum cascade lasers

XIAOQIONG QI,[1] KARL BERTLING,[1] MITCHELL S. STARK,[2] THOMAS TAIMRE,[3] YUNG-CHING KAO,[2] YAH LENG LIM,[1] SHE HAN,[1] BLAKE O'BRIEN,[4] ANGUS COLLINS,[4], MICHAEL WALSH,[4] JARI TORNIAINEN,[1] TIMOTHY GILLESPIE,[1] BOGDAN C. DONOSE,[1] PAUL DEAN,[5] LIAN HE LI,[5] EDMUND H. LINFIELD,[5] A. GILES DAVIES,[5] DRAGAN INDJIN,[5] H. PETER SOYER,[2,6] AND ALEKSANDAR D. RAKIĆ[1,*]

[1]*School of Information Technology and Electrical Engineering, The University of Queensland, Brisbane, QLD 4072 Australia*
[2]*The University of Queensland Diamantina Institute, The University of Queensland, Dermatology Research Centre, Brisbane, QLD, 4102, Australia*
[3]*School of Mathematics and Physics, The University of Queensland, Brisbane, QLD 4072 Australia*
[4]*Sullivan Nicolaides Pathology, Brisbane, QLD, Australia*
[5]*School of Electronic and Electrical Engineering, University of Leeds, Leeds LS2 9JT UK*
[6]*Department of Dermatology, Princess Alexandra Hospital, Brisbane, Queensland, Australia*
[*]*rakic@itee.uq.edu.au*

Two benign naevus blocks from Patient 1 and 2 with the information listed in Table 1 of the main manuscript were used to determine the best experimental protocol of sectioning thickness for THz imaging employed QCLs under LFI technology. The experimental design to obtain the best imaging protocol for sample sectioning and the associated results are shown in the following sections.

1. Experiment design to obtain the best imaging protocol for sample sectioning

Due to the precious of human tissue samples, we need to find the minimum slice thickness sectioned from the PFPE/FFPE blocks that still provides good contrast between skin pathology and healthy skin. In order to determine the best experimental protocol working for THz imaging based on QCL in a LFI system, we sectioned the tissue block from Patient 1 into 5, 10, 20, 50, 100, and 200 µm thick slices, and compared the correlation with the adjacent sectioned and stained H&E images, it was found the minimum thickness that still give us good contrast is between 20 µm to 50 µm. Therefore, we sectioned the tissue block from Patient 2 into 20, 30, 40, and 50 µm to further determine accurately the thinnest slice with good contrast in THz images. The THz amplitude and phase images for the section with the thickness of 30, 40, and 50 µm are shown in Fig. 1(a), (b), (c), respectively, where (a1), (b1), and (c1) are THz amplitude images; (a2), (b2), and (c2) are THz phase images (20 µm-thick section give us very blurry images thus not shown). It can be observed that good contrast between paraffin, epidermis layer, and further dermis layer can be obtained from the THz amplitude and phase images of the 50 µm-thick sample in Fig 1 (c1) and (c2). We can see clearly the boundary between the sample and the surrounding paraffin from THz images of the 50 µm-thick sample and there are also much more detailed contrast inside this sample slice. However, we can only see part of the boundary from the 30 and 40 µm-thick samples [Fig 1 (a) and (b)]. In particular, for the THz phase images, inside the sample image the contrast is dominant by the paraffin. It is found that the THz image quality for the sections of 100 µm-thick and above is the same as 50 µm-thick sections, however, thick sections above 100 µm leads to a curving of the surface when they are applied on the slides. Therefore, 50 µm is the best thickness of the dehydrated FFPE or PFPE section we identified that

can provide THz contrast.

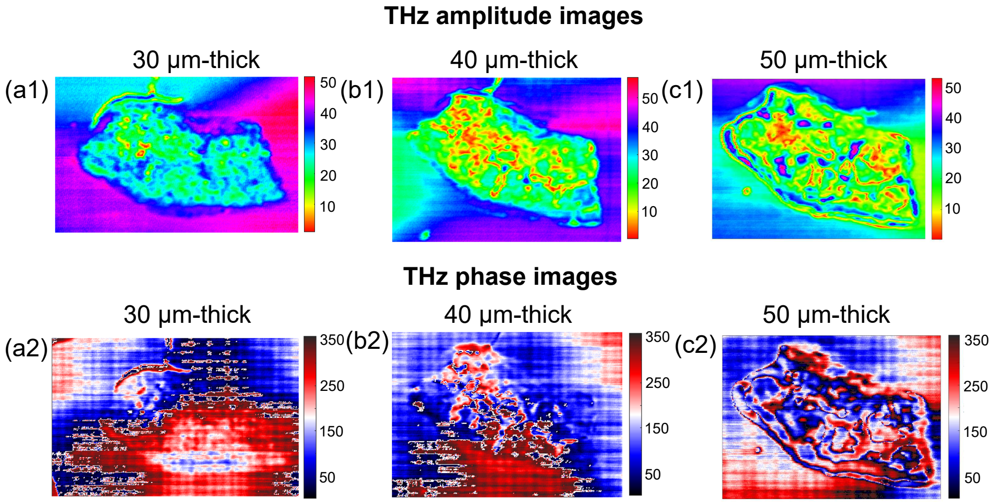

Fig. 1. Comparison of THz images of sectioned slices from Patient 1 block with the thickness of (a) 30 μm, (b) 40 μm, and (c) 50 μm. (a1), (b1), and (c1) are THz amplitude images; (a2), (b2), and (c2) are THz phase images.

2. Experimental results for human skin samples used for testing

In this section we provide THz imaging results for the two benign naevus samples from Patient 1 and 2 that used to identify the best experimental protocol for THz imaging.

Figure 2 depicts comparisons between images for the 50 μm benign naevus tissue sample from Patient 1. From the microscope photo of the tissue shown in Fig. 2(a), it is hard to see any contrast inside this sample. However, there are stained benign naevus cells as marked by the green box in Fig. 2(b), along with a marked normal skin area in the blue box. The THz amplitude and phase image, which represent the amplitude and the phase of Fourier transform of the self-mixing signal at the peak frequency at each pixel is shown in Fig. 2(c) and Fig. 2(d), respectively. The tissue outlined in the THz images is very similar to that in its pathology image as we overlapped them in Fig. 2(e). It should be noted that the outermost green layer in the THz amplitude image indicates the air bubbles around the skin slice when it was loaded onto the polystyrene slide, which can be clearly observed from the microscope photo. The THz images can distinguish epidermis (blue and purple layer) and dermis region (most green) of the sample, where the epidermis tissue has higher reflection compared to the dermis layer. This is due to much higher contrast of the properties between the epidermis and the polystyrene slide compared with those between the dermis (comprised of nerves and capillaries which epidermis does not contain) and polystyrene. Furthermore, after finding the corresponding pathology and normal skin region in the THz images in Fig. 2(c), the benign naevus cell region in the THz amplitude image (marked in the green box) has slightly higher reflection than the normal skin (marked by the blue box), which suggests that the naevus cells absorb less THz radiation at this emission frequency (2.8 THz) than the other tissue regions. The phase contrast between the epidermis and dermis layer is dominant in the THz phase image in Fig. 2(d). The area outside of the tissue section in the THz images in Fig. 2(c) and (d) is paraffin. THz images provide more features than the microscope photo due to different THz properties of the tissues in each of the tissue regions. In order to qualitatively describe the THz contrast between the pathology and normal skin, the density distribution of the pixels in the marked green and blue boxes were used to

find the clustering of the benign naevus cells and the normal skin in the THz amplitude–phase map, as shown in Fig. 2(e). The blue and green clouds indicate the density distributions of the normal skin and benign naevus respectively, together with the centroid for each group indicated by orange triangle and black cross, respectively. Although the two regions share the same phase range, the normalized amplitude of benign naevus cells is higher (centroid: 1.121) than that of the normal skin (centroid: 0.9992).

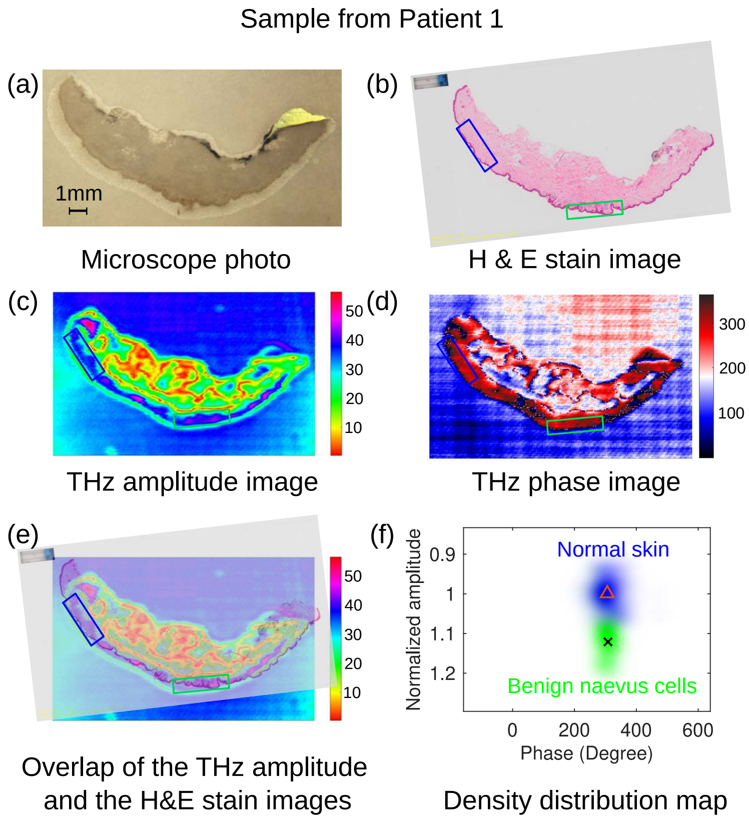

Fig. 2. Comparison of images for the benign naevus tissue from Patient 1: (a) Microscope photo of the sample; (b) H&E stain image; (c) THz amplitude image; (d) THz phase image; (e) Overlap of the THz amplitude and the H&E stain images; (f) Density distribution of the point cloud of the normal skin marked by the blue box (blue cloud) and the benign naevus marked by the green box (green cloud), along with the centroid for each group in the THz amplitude–phase map:(306.0°, 0.9992) and (308.3°, 1.121) for the normal skin and benign naevus region, respectively. The marked region in green and blue boxes in (b) to (e) indicate the benign naevus cell region and normal skin region, respectively

Similar analysis was performed for another benign naevus sample from Patient 2 with the results shown in Fig. 3. The microscope photo and the H&E stain image with benign congential naevus cells below epidermis in the green box and the normal skin region in the blue box of the tissue slice are shown in (a) and (b), respectively. THz contrast between the naevus cells and normal skin can be observed both in the amplitude and phase image in Fig. 3(c) and (d). The density distribution map for the pixels in the skin region in the marked boxes further verifies the separation of THz imaging on benign naevus cells and normal skin in both normalized amplitude and phase in in Fig. 3 (f). There is partially overlap between the normal skin and benign naevus

region in the amplitude–phase map, which reflects some of the pixels in the marked two regions in (c) and (d) have similar amplitude and phase. However, the centroids that indicates the highest density amplitude for the benign naevus is still 1.39 times higher than that for the normal skin (1.525 and 1.099, respectively), and the phase centroids are also seperated by 95.7°. The results from both of the two benign naevus tissue from Patient 1 and 2 agrees with each other that the benign cell region has higher reflection than the normal skin.

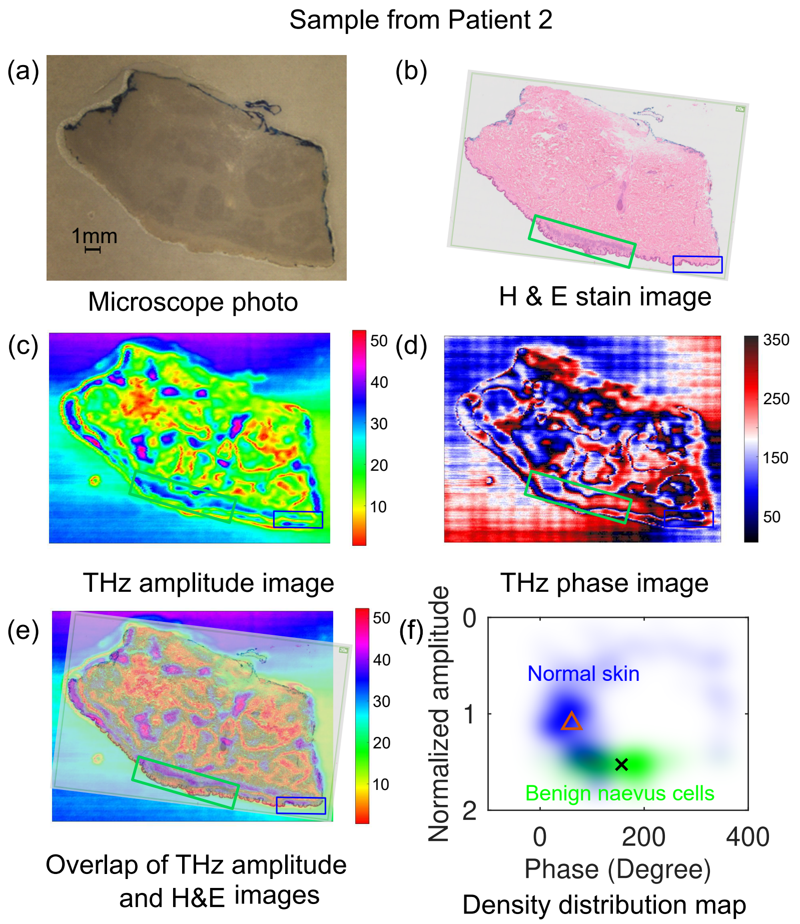

Fig. 3. Comparison of images for the skin sample that contains benign congential naevus cells from Patient 2: (a) Microscope photo of the sample; (b) H&E stain image; (c) THz amplitude image; (d) THz phase image; (e) Overlap of the THz amplitude and H&E images; (f) Density distribution of the normal skin and benign naevus in the THz amplitude and phase map, along with the centroids in each clustering marked by the triangle and cross: (60.77°, 1.099) for normal skin and (156.5°, 1.525) for benign naevus region. The marked region in green and blue boxes in (b) to (e) indicate the benign naevus cell region and normal skin region, respectively.
